# Intermediate Uveitis as the Initial and Only Presentation of Syphilis

**DOI:** 10.4274/tjo.galenos.2019.72558

**Published:** 2019-10-24

**Authors:** Sevcan Yıldız Balcı, Ece Turan Vural, Şehnaz Özçalışkan

**Affiliations:** 1 University of Health Sciences, Haydarpaşa Numune Training and Research Hospital, Ophthalmology Clinic, İstanbul, Turkey; 2 University of Health Sciences, Beyoğlu Eye Training and Research Hospital, Ophthalmology Clinic, İstanbul, Turkey

**Keywords:** Syphilis, intermediate uveitis, cystoid macular edema

## Abstract

We report a patient with unilateral syphilitic intermediate uveitis without dermatological, neurological, or any systemic involvement. He presented to our clinic with complaints of eye floaters and worsening visual acuity in the left eye. He had intermediate uveitis and cystoid macular edema in that eye and both venereal disease research laboratory and microhemagglutination assay for *Treponema pallidum* serological tests were confirmatory for syphilis. Ocular manifestations of syphilis have variable presentations, and it should be considered when diagnosing unexplained ocular inflammatory diseases, even if the patient’s recent history and systemic evaluation are not compatible.

## Introduction

Syphilis is a sexually transmitted infectious disease caused by *Treponema pallidum*.^1^ Syphilis progresses through three stages: primary, secondary, and tertiary (late-stage).^2^ Ocular involvement is rare in the primary stage and mainly presents as chancres of the eyelids and conjunctiva. In secondary syphilis (after 6-8 weeks), patients develop the symptoms of influenza, arthralgia, myalgia, headache, sore throat, lymphadenopathy, fever, and maculopapular skin rashes, especially on the palms and soles. After the latent period of the disease, which follows the secondary stage and ranges from 1 year to decades, tertiary syphilis starts. In the tertiary stage, patients develop cardiovascular syphilis and neurosyphilis as well as granulomatous lesions called gumma, which can be seen in the iris and choroid.^3^ Syphilitic patients can present with granulomatous or nongranulomatous uveitis. Focal or multifocal chorioretinitis, usually associated with a variable degree of vitritis, is the most common finding, and placoid chorioretinitis in the macula is the pathognomonic finding in syphilitic uveitis. Neuro-ophthalmic symptoms include oculomotor nerve paralysis, optic neuropathy, and retrobulbar neuritis, which are seen in tertiary syphilis and neurosyphilis. Although syphilis is considered to be responsible for only 1-2% of all uveitis cases, it should be noted that it is a great masquerader and should be considered in case of any kind of intraocular inflammation.^4^ Recently, patients diagnosed with ocular syphilis were associated with coinfections such as human immunodeficiency virus (HIV). Here, we report a syphilis case that presented with unilateral intermediate uveitis (IMU) with no other systemic findings in an HIV-negative patient.

## Case Report

**Figure 1:**
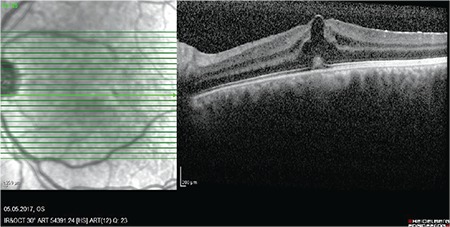
Optical coherence tomography showing cystoid macular edema in the left eye

**Figure 2:**
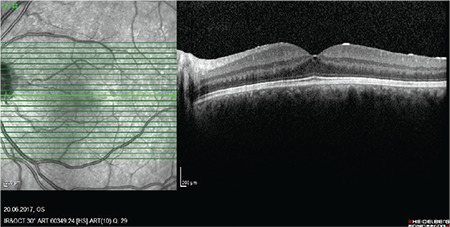
Optical coherence tomography showing regression of cystoid macular edema at week 3

A 22-year-old man presented with a long history of floaters in his left eye. Visual acuity of the right eye was 10/10 and that of the left eye was 7/10. Intraocular pressure was measured as 11/12 mmHg. Slit-lamp and fundus examinations of the right eye were normal, while slit-lamp examination of the left eye showed +2 cells in the vitreous. Fundus examination showed minimal hyperemia in the optic disc. Fundus fluorescein angiography revealed a normal right eye and focal leakage in the macula of the left eye as well as fluorescein leakage in the optic disc. Optical coherence tomography (OCT) demonstrated cystoid macular edema in the left eye (Figure 1). The patient reported a history of sexual promiscuity. His laboratory tests showed normal results for complete blood count, liver function tests, and blood urea nitrogen. Toxoplasma and HIV immunoglobulin G (IgG) and IgM tests were negative. He had elevated C-reactive protein, erythrocyte sedimentation rate of 54 mm/h, and negative purified protein derivative  test. His chest radiography and brain magnetic resonance imaging were normal. As the results of the venereal disease research laboratory (VDRL) and *T. pallidum* hemagglutination tests were positive, the patient was diagnosed with ocular syphilis. In consultation with the Department of Infectious Diseases, the patient was evaluated for systemic infectious diseases and there was no evidence of past or current dermatological, neurological, or systemic involvement of the disease. The patient underwent a lumbar puncture and VDRL test of the cerebrospinal fluid was negative. The patient was treated with intravenous ceftriaxone 2 g/day for 14 days because he had allergy to penicillin. In addition, 1 mg/kg/day oral methylprednisolone was added after 48 hours of treatment and was discontinued 2 days before the antibiotherapy. Improvement in the patient’s clinical symptoms was observed after 3 weeks of therapy and the patient’s condition was stable at 6-month control examination. Visual acuity of the left eye was 10/10, vitreous cells were negative, and optic disc and macula were normal. OCT showed regression of the cystoid macular edema (Figure 2). At 12 months, we did not observe any systemic involvement of infectious disease and repeated laboratory tests including *Toxoplasma* and HIV IgG and IgM were negative.

## Discussion

In this article, we aimed to present a case of syphilis that had only ocular symptoms without any dermatological, neurological, or systemic findings. Syphilis can involve any segment or layer of the eye. The ophthalmologic manifestations of syphilis include uveitis, retinitis, scleritis, vitritis, retinal vasculitis, optic nerve involvement, and papillary abnormalities. Ocular involvement in syphilis mainly occurs in the secondary and tertiary stages.^5^ In a review analyzing the data of 143 patients with syphilitic uveitis, 55.2% of the patients had posterior uveitis, 25.2% had panuveitis and 19.6% had anterior or intermediate uveitis.^6^ Anshu et al.^7^ found in their study that nongranulomatous anterior uveitis was a more frequent presentation in syphilitic uveitis.

Guidelines from Europe (International Union against Sexually Transmitted Infections) and the United States (Centers for Disease Control and Prevention [CDC]) recommend the standard use of intravenous benzyl penicillin at a dose of 12-24 million units (MU) per day, with 3-4 MU given every 4 hours for 10-21 days.^8,9^ The recent World Health Organization Sexually Transmitted Infection guidelines recommend benzathine penicillin G administered intramuscularly at a dose of 2.4 MU once weekly for 3 consecutive weeks to treat late syphilis (including ocular syphilis).^10^ In case of neurosyphilis, however, 12-24 MU/day crystalline penicillin G should be administered as intravenous 2-4 MU every 4 hours for 10-14 days.^11^ Cases with ocular involvement should be treated as those with neurosyphilis. As immunological reactions are also believed to be involved in the pathogenesis of late syphilis, it seems reasonable to administer corticosteroids in combination with standard antibacterial regimens to treat syphilitic uveitis.^12^ Patients with penicillin allergy should be treated with ceftriaxone 2 g daily intramuscular or intravenously for 10-14 days.^13^

In recent years, there has been an increase in the incidence of syphilis, which causes various types of ocular involvement.^14^ Jones^15^ reviewed 3000 new uveitic cases and found that the incidence of syphilitic uveitis was <1%. Sahin and Ziaei^16^ found that 1.07% of uveitic patients in Turkey were diagnosed with ocular syphilis. In another recent study from Turkey, Yalçındağ et al.^17^ analyzed a nationwide web-based registry of patients (4863) with uveitis and reported that syphilitic uveitis was diagnosed in 5 cases (0.1%).

The CDC reported that there is an increased risk of all primary and secondary syphilis cases occurred in men who have sex with men and rise in incidence of ocular syphilis patient who is co-infected with HIV.^18,19^

Although our patient did not show systemic symptoms specific to syphilis at the time of admission, we were able to diagnose ocular syphilis thorough a detailed anamnesis and ensured that he received appropriate treatment. Medical history-taking has a significant role in diagnosis. As the ocular symptoms of the disease can be seen at any stage and may be the initial symptoms in some cases, clinical manifestations of syphilis in the eye are similar to many other infectious uveitic diseases. Therefore, syphilis should be considered for all ocular inflammatory conditions in patients with a history of risky sex, even in the absence of any other clinical symptoms of primary or secondary syphilis, and they should be followed long-term for syphilis reinfection and HIV coinfection.
